# *N*-Hydroxy-*N*-methyl­ammonium chloride

**DOI:** 10.1107/S160053680801355X

**Published:** 2008-05-10

**Authors:** Seik Weng Ng

**Affiliations:** a Department of Chemistry, University of Malaya, 50603 Kuala Lumpur, Malaysia

## Abstract

In the crystal structure of the title compound, CH_6_NO^+^·Cl^−^, the cations and anions are linked by N–H⋯Cl and O–H⋯Cl hydrogen bonds into an undulating layer motif [Schläfli symbol: 4(8).6(8).8(2)]. All non-H atoms lie on a mirror plane.

## Related literature

Only the cell dimensions of *N*-methyhydroxy­lammonium chloride have hitherto been reported; see: Toft & Jerslev (1967 ▶).
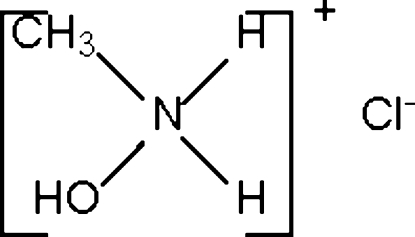


## Experimental

### 

#### Crystal data

CH_6_NO^+^·Cl^−^
*M* *_r_* = 83.52
Orthorhombic, 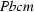

*a* = 7.8084 (3) Å
*b* = 8.7109 (3) Å
*c* = 6.0232 (1) Å
*V* = 409.69 (2) Å^3^
*Z* = 4
Mo *K*α radiation
μ = 0.73 mm^−1^
*T* = 100 (2) K
0.25 × 0.20 × 0.15 mm

#### Data collection

Bruker SMART APEX diffractometer
Absorption correction: multi-scan (*SADABS*; Sheldrick, 1996 ▶) *T*_min_ = 0.839, *T*_max_ = 0.899
3330 measured reflections
558 independent reflections
493 reflections with *I* > 2σ(*I*)
*R*_int_ = 0.029

#### Refinement

*R*[*F* ^2^ > 2σ(*F* ^2^)] = 0.024
*wR*(*F* ^2^) = 0.070
*S* = 1.07
558 reflections
40 parameters
6 restraints
All H-atom parameters refined
Δρ_max_ = 0.28 e Å^−3^
Δρ_min_ = −0.26 e Å^−3^

### 

Data collection: *APEX2* (Bruker, 2007 ▶); cell refinement: *SAINT* (Bruker, 2007 ▶); data reduction: *SAINT*; program(s) used to solve structure: *SHELXS97* (Sheldrick, 2008 ▶); program(s) used to refine structure: *SHELXL97* (Sheldrick, 2008 ▶); molecular graphics: *X-SEED* (Barbour, 2001 ▶); *OLEX* (Dolomanov *et al.*, 2003 ▶); software used to prepare material for publication: *publCIF* (Westrip, 2008 ▶).

## Supplementary Material

Crystal structure: contains datablocks global, I. DOI: 10.1107/S160053680801355X/tk2270sup1.cif

Structure factors: contains datablocks I. DOI: 10.1107/S160053680801355X/tk2270Isup2.hkl

Additional supplementary materials:  crystallographic information; 3D view; checkCIF report

## Figures and Tables

**Table 1:** Hydrogen-bond geometry (Å, °)

*D*—H⋯*A*	*D*—H	H⋯*A*	*D*⋯*A*	*D*—H⋯*A*
O—H1⋯Cl	0.84 (1)	2.16 (1)	2.998 (1)	171 (2)
N—H2⋯Cl^i^	0.88 (1)	2.33 (1)	3.1241 (4)	149 (1)

Symmetry code: (i) 
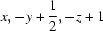
.

**Fig. 1.**
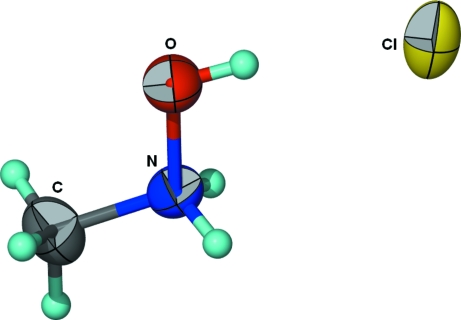
Thermal ellipsoid plot (Barbour, 2001) of N-methylhydroxylammonium chloride at the 70% probability level.

**Fig. 2.**
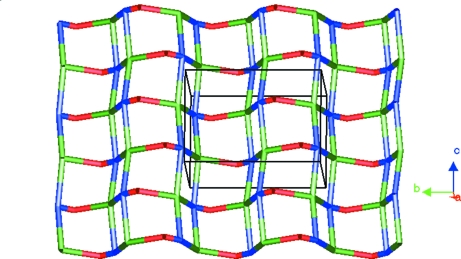
OLEX (Dolomanov et al., 2003) representation of the hydrogen-bonded layer structure.

## supplementary crystallographic information

### Comment

We are interested in the crystal structures of small organic molecules, molecules whose asymmetric unit consist of four or five non-hydrogen atoms only. *N*-Methylhydroxylammonium chloride (Scheme I) provides an example of such a system. However, the crystal structure is not known with only unit-cell dimensions reported (Toft & Jerslev, 1967).

The structure (Fig. 1) consists of cations and anions that are linked by N–H···Cl and O–H···Cl hydrogen bonds into an undulating layer motif [Schläfli symbol: 4(8).6(8).8(2)], Fig. 2 & Table 1. The non-hydrogen atoms lie on a mirror plane.

### Experimental

The chemical as purchased from the Aldrich Chemical Company was crystalline.

### Refinement

All hydrogen atoms were located in a difference Fouier map, and were refined with distance restraints (C–H 0.99±0.01, N–H 0.88±0.01 and O–H 0.84±0.01 Å). For the methyl group, an additional H···H = 1.59±0.01 Å was imposed. The temperature factors were freely refined.

### Crystal data

**Table:**

CH_6_NO^+^·Cl^–^	*F*_000_ = 176
*M**_r_* = 83.52	*D*_x_ = 1.354 Mg m^−^^3^
Orthorhombic, *P**b**c**m*	Mo *K*α radiation λ = 0.71073 Å
Hall symbol: -P 2c 2b	Cell parameters from 1402 reflections
*a* = 7.8084 (3) Å	θ = 2.6–28.2º
*b* = 8.7109 (3) Å	µ = 0.73 mm^−^^1^
*c* = 6.0232 (1) Å	*T* = 100 (2) K
*V* = 409.69 (2) Å^3^	Prism, colorless
*Z* = 4	0.25 × 0.20 × 0.15 mm

### Data collection

**Table:**

Bruker SMART APEX diffractometer	558 independent reflections
Radiation source: fine-focus sealed tube	493 reflections with *I* > 2σ(*I*)
Monochromator: graphite	*R*_int_ = 0.029
*T* = 100(2) K	θ_max_ = 28.4º
ω scans	θ_min_ = 2.6º
Absorption correction: Multi-scan(SADABS; Sheldrick, 1996)	*h* = −10→8
*T*_min_ = 0.839, *T*_max_ = 0.899	*k* = −11→11
3330 measured reflections	*l* = −8→8

### Refinement

**Table:**

Refinement on *F*^2^	Secondary atom site location: difference Fourier map
Least-squares matrix: full	Hydrogen site location: inferred from neighbouring sites
*R*[*F*^2^ > 2σ(*F*^2^)] = 0.024	All H-atom parameters refined
*wR*(*F*^2^) = 0.070	*w* = 1/[σ^2^(*F*_o_^2^) + (0.0402*P*)^2^ + 0.0529*P*] where *P* = (*F*_o_^2^ + 2*F*_c_^2^)/3
*S* = 1.07	(Δ/σ)_max_ = 0.001
558 reflections	Δρ_max_ = 0.28 e Å^−^^3^
40 parameters	Δρ_min_ = −0.26 e Å^−^^3^
6 restraints	Extinction correction: SHELXL, Fc^*^=kFc[1+0.001xFc^2^λ^3^/sin(2θ)]^-1/4^
Primary atom site location: structure-invariant direct methods	Extinction coefficient: 0.15 (1)

### Fractional atomic coordinates and isotropic or equivalent isotropic displacement parameters (Å^2^)

**Table:**

	*x*	*y*	*z*	*U*_iso_*/*U*_eq_	
Cl	0.35777 (6)	0.42790 (6)	0.2500	0.0433 (2)	
O	0.1297 (2)	0.1511 (2)	0.2500	0.0389 (3)	
N	0.2586 (2)	0.0376 (2)	0.2500	0.0300 (3)	
C	0.1739 (3)	−0.1127 (3)	0.2500	0.0457 (5)	
H1	0.184 (3)	0.2349 (17)	0.2500	0.048 (6)*	
H2	0.3265 (17)	0.0482 (17)	0.366 (2)	0.041 (4)*	
H3	0.2644 (18)	−0.190 (2)	0.2500	0.048 (6)*	
H4	0.1075 (14)	−0.121 (2)	0.1170 (8)	0.064 (5)*	

### Atomic displacement parameters (Å^2^)

**Table:**

	*U*^11^	*U*^22^	*U*^33^	*U*^12^	*U*^13^	*U*^23^
Cl	0.0455 (3)	0.0591 (4)	0.0254 (3)	−0.0196 (2)	0.000	0.000
O	0.0315 (7)	0.0324 (7)	0.0529 (8)	0.0032 (5)	0.000	0.000
N	0.0282 (6)	0.0336 (7)	0.0282 (7)	0.0016 (5)	0.000	0.000
C	0.053 (1)	0.033 (1)	0.051 (1)	−0.004 (1)	0.000	0.000

### Geometric parameters (Å, °)

**Table:**

O—N	1.411 (2)	N—H2	0.88 (1)
N—C	1.467 (3)	C—H3	0.98 (1)
O—H1	0.84 (1)	C—H4	0.96 (1)
			
N—O—H1	104.5 (16)	N—C—H3	107.0 (12)
O—N—C	107.70 (14)	N—C—H4	108.0 (11)
O—N—H2	110.8 (10)	H3—C—H4	110.0 (9)
C—N—H2	111.3 (10)		

### Hydrogen-bond geometry (Å, °)

**Table:**

*D*—H···*A*	*D*—H	H···*A*	*D*···*A*	*D*—H···*A*
O—H1···Cl	0.84 (1)	2.16 (1)	2.998 (1)	171 (2)
N—H2···Cl^i^	0.88 (1)	2.33 (1)	3.1241 (4)	149 (1)

Symmetry codes: (i) *x*, −*y*+1/2, −*z*+1.
